# Deficiency of Vasodilator-Stimulated Phosphoprotein (VASP) Increases Blood-Brain-Barrier Damage and Edema Formation after Ischemic Stroke in Mice

**DOI:** 10.1371/journal.pone.0015106

**Published:** 2010-12-03

**Authors:** Peter Kraft, Peter Michael Benz, Madeleine Austinat, Marc Elmar Brede, Kai Schuh, Ulrich Walter, Guido Stoll, Christoph Kleinschnitz

**Affiliations:** 1 Department of Neurology, University of Würzburg, Würzburg, Germany; 2 Institute for Clinical Biochemistry and Pathobiochemistry, University of Würzburg, Würzburg, Germany; 3 Department of Physiology, University of Würzburg, Würzburg, Germany; 4 Department of Anesthesiology, University of Würzburg, Würzburg, Germany; University of Muenster, Germany

## Abstract

**Background:**

Stroke-induced brain edema formation is a frequent cause of secondary infarct growth and deterioration of neurological function. The molecular mechanisms underlying edema formation after stroke are largely unknown. Vasodilator-stimulated phosphoprotein (VASP) is an important regulator of actin dynamics and stabilizes endothelial barriers through interaction with cell-cell contacts and focal adhesion sites. Hypoxia has been shown to foster vascular leakage by downregulation of VASP *in vitro* but the significance of VASP for regulating vascular permeability in the hypoxic brain *in vivo* awaits clarification.

**Methodology/Principal Findings:**

Focal cerebral ischemia was induced in *Vasp^−/−^* mice and wild-type (WT) littermates by transient middle cerebral artery occlusion (tMCAO). Evan's Blue tracer was applied to visualize the extent of blood-brain-barrier (BBB) damage. Brain edema formation and infarct volumes were calculated from 2,3,5-triphenyltetrazolium chloride (TTC)-stained brain slices. Both mouse groups were carefully controlled for anatomical and physiological parameters relevant for edema formation and stroke outcome. BBB damage (p<0.05) and edema volumes (1.7 mm^3^±0.5 mm^3^ versus 0.8 mm^3^±0.4 mm^3^; p<0.0001) were significantly enhanced in *Vasp^−/−^* mice compared to controls on day 1 after tMCAO. This was accompanied by a significant increase in infarct size (56.1 mm^3^±17.3 mm^3^ versus 39.3 mm^3^±10.7 mm^3^, respectively; p<0.01) and a non significant trend (p>0.05) towards worse neurological outcomes.

**Conclusion:**

Our study identifies VASP as critical regulator of BBB maintenance during acute ischemic stroke. Therapeutic modulation of VASP or VASP-dependent signalling pathways could become a novel strategy to combat excessive edema formation in ischemic brain damage.

## Introduction

Disruption of the blood-brain barrier (BBB) and successive edema formation are pathological hallmarks of many neurological diseases and can dramatically deteriorate clinical symptoms especially in patients with ischemic stroke [1], [2]. So far no medication, e. g. steroids or hyperosmolaric solutions, has proven to effectively reduce brain edema in acute stroke [3]–[5] and the molecular mechanisms underlying edema formation are largely unknown.

The vascular endothelium controls the transition of fluids and cells between blood vessels and the interstitium of most organs including the brain [6]. Efficient barrier function requires stable cell-cell- and cell-matrix-interactions and paracellular permeability is regulated by a complex interplay of transmembrane adhesion molecules, tight junctions and cytoskeletal proteins [7]–[10]. Impairment of any of these interactions can increase endothelial leakage and result in excessive edema formation [9]. Tight junctions represent the most apical of these cell-cell contacts and are of major importance for sealing vascular barriers. While earlier studies mainly focused on the relevance of transmembrane adhesion molecules for tight junction functionality, there is a growing body of evidence pointing towards a critical role of actin cytoskeleton dynamics in modulating the permeability of the BBB [11] and other endothelia [12], [13].

Vasodilator-stimulated phosphoprotein (VASP) is the founding member of the Enabled/vasodilator-stimulated phosphoprotein (Ena/VASP) protein family [14]. In mammals, this family comprises three molecules: mammalian Ena (Mena), VASP, and Ena/VASP-like (EVL). Ena/VASP proteins are important mediators in actin cytoskeleton control and participate in a variety of actin-based processes such as cell-adhesion, -spreading, and -shape change [14]. Only recently the fundamental role of VASP for maintaining endothelial barrier functions has been established. Several *in vitro* studies verified the expression of VASP at endothelial cell-cell and cell-matrix contacs in different vascular beds [15]–[19]. Moreover, VASP was shown to initiate perijunctional actin filament assembly thereby stabilizing cell-cell contacts and decreasing endothelial permeability [18]. Accordingly, increased vascular leakage was observed in the inflamed skin of *Vasp^−/−^* mice [18] and mice deficient in *Mena*, *Vasp*, and *EVL* develop spontaneous and generalized edema due to severe vessel texture defects [20]. Although VASP is expressed in primary brain capillary endothelial cells (BCECs) [21], little is known about the significance of VASP for regulating vascular permeability in the central nervous system (CNS) especially under pathological conditions *in vivo*.

We here show that BBB leakage and edema formation are increased in *Vasp^−/−^* mice in a model of brain ischemia/reperfusion(I/R)-injury.

## Results

### Systemic blood pressure, cerebral blood flow and the brain vasculature are unchanged in *Vasp^−/−^* mice

**Figure 1:**
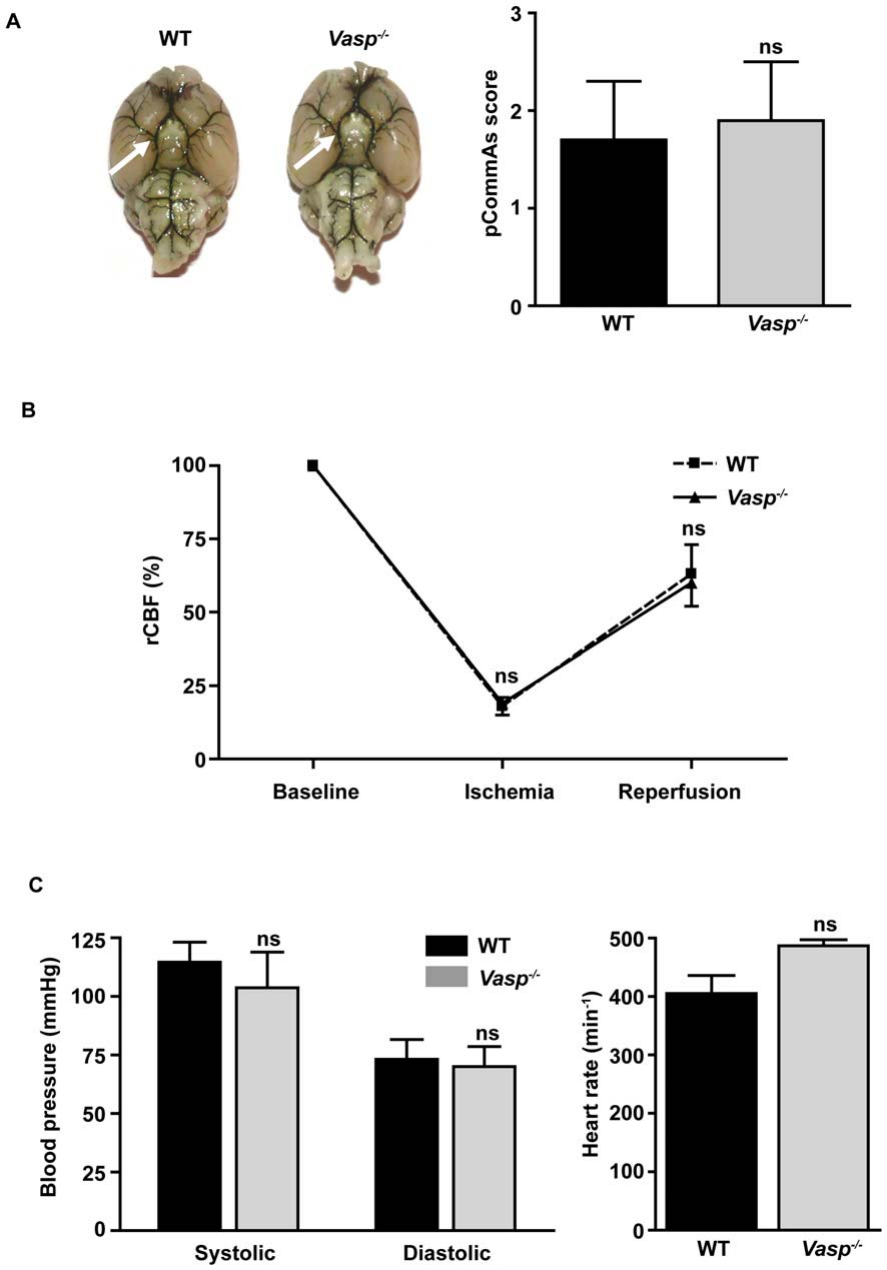
*Vasp* deficiency does not alter anatomical and physiological parameters relevant for stroke outcome. (**A**) (left) A complete Circle of Willis (arrows) was present in wild-type (WT) and *Vasp^−/−^* mice and the trunk and branches of the middle cerebral artery (MCA) were similar in both groups as depicted by ink perfusion. (right) The formation of the posterior communicating arteries (PComAs) was quantitatively assessed under a microscope in both mouse groups. The PComAs score did not differ between *Vasp^−/−^* mice and WT controls (n = 5/group), p>0.05; unpaired, two-tailed Student's t-test compared with WT mice. (**B**) rCBF in the MCA territory was measured by Laser Doppler flowmetry before (baseline) and immediately after MCAO (ischemia), and again 10 min after removal of the occluding filament (reperfusion). No significant differences in rCBF were observed at any time point between WT and *Vasp^−/−^* mice (n = 5/group); p>0.05. Bonferroni-corrected 2-way ANOVA compared to baseline rCBF. (**C**) Systolic and diastolic blood pressure (RR) (left) as well as heart rates (right) are similar in *Vasp^−/−^* mice and WT controls, p>0.05; unpaired, two-tailed Student's t-test compared with WT mice. ns: not significant.

First of all we collected critical physiological and anatomical parameters that could possibly influence stroke outcome and edema formation in genetically altered *Vasp^−/−^* mice. A complete Circle of Willis was identified in *Vasp^−/−^* and *Vasp^+/+^* mice upon macroscopic assessment and the distribution of the middle cerebral artery (MCA) trunk and branches appeared to be identical (Figure 1A, left panel). Collateralisation via the posterior communicating arteries (PComAs) can affect the susceptibility for brain ischemia in transgenic mice [22]. We therefore assessed the development of PComAs in *Vasp^−/−^* mice and littermate controls using a quantitative score [23]. No differences in PComAs scores were found between the both groups (1.9±0.6 versus 1.7±0.6; p>0.05) (Figure 1A, right panel).

In the present study tMCAO was used to induce focal brain ischemia. After advancing the filament to the origin of the MCA the decrease in regional cerebral blood flow (rCBF) was similar between WT mice and *Vasp^−/−^* mice (17.7%±3.2% versus 18.7%±4.0%; p>0.05) (Figure 1B). Ten minutes after removal of the filament (reperfusion) rCBF in the MCA territory was reconstituted to >60% of baseline levels and again did not significantly differ between the two mouse groups (62.7%±10.4% versus 60.3%±8.3%; p>0.05) (Figure 1B). These findings exclude preformed alterations in rCBF related to the *Vasp^−/−^* genotype and prove that MCA occlusion and reperfusion were sufficient in our model.

Changes in arterial blood pressure can directly influence final stroke sizes and the magnitude of BBB disruption [24]. We therefore compared systemic blood pressure and heart rate between the two groups. Again, no significant differences were observed (systolic blood pressure: 115 mm Hg ±9 mm Hg versus 104 mm Hg ±15 mm Hg; diastolic blood pressure 73 mm Hg ±8 mm Hg versus 70 mm Hg ±9 mm Hg; heart rate: 405 min^−1^ ±31 min^−1^ versus 487 min^−1^ ±11 min^−1^; p>0.05) (Figure 1C).

### *Vasp* deficiency increases infarct size, blood-brain-barrier damage and edema formation after ischemic stroke

**Figure 2:**
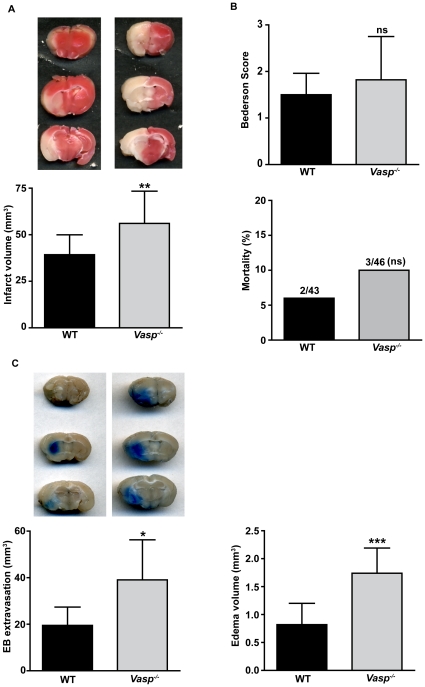
*Vasp* deficiency increases infarct volumes, BBB damage and edema formation after ischemic stroke. (**A**) (top) Representative 2,3,5-triphenyltetrazolium chloride (TTC) stains of three corresponding coronal brain sections of wild-type (WT) and *Vasp^−/−^* mice on day 1 after tMCAO. The ischemic infarctions appear white. (bottom) Brain infarct volumes as measured by planimetry without correction for edema (direct volumes) in WT (n = 15) and *Vasp^−/−^* mice (n = 17) on day 1 after tMCAO, **p<0.01; unpaired, two-tailed Student's t-test compared with WT mice. (**B**) Neurological Bederson score (top) and mortality rates (bottom) of WT mice (n = 15) and *Vasp^−/−^* mice (n = 17) on day 1 after tMCAO, p>0.05; non-parametric Mann Whitney test (for Bederson score) or Fisher̀s exact contingency test (for mortality) compared with WT mice. (**C**) (top, left) Representative coronal brain sections from *Vasp^−/−^* and WT mice on day 1 after tMCAO and injection of the vascular tracer Evan's blue. (bottom, left) Volume of Evan's blue (EB) extravasation as determined by planimetry (n = 5/group). (right) Brain edema volumes as calculated from direct and indirect infarct volumes on day 1 after tMCAO in WT mice (n = 15) and *Vasp^−/−^* mice (n = 17), *p<0.05, ***p<0.0001; unpaired, two-tailed Student's t-test compared with WT mice.

We next subjected *Vasp^−/−^* mice to tMCAO and, after 24 h, assessed infarct volumes by staining brain sections with 2,3,5-triphenyltetrazolium chloride (TTC) (Figure 2A, upper panel). Infarct volumes were significantly larger, by approximately 45%, in *Vasp*-deficient mice than in WT controls (56.1 mm^3^±17.3 mm^3^ versus 39.3 mm^3^±10.7 mm^3^, respectively; p<0.001) (Figure 2A, lower panel). Although mice without *Vasp* tended to develop more severe neurological deficits after stroke, the difference was not statistically significant (Bederson score: 1.8±0.9 versus 1.5±0.5, respectively; p>0.05) (Figure 2B, upper panel). In line with these results, mortality rates were similar in both groups (p>0.05) (Figure 2B, lower panel). Thus, our observations corroborate previous reports on a poor correlation between infarct size and neurological outcome in rodents [25]–[27].

Next we sought to elucidate the underlying mechanisms of this VASP-specific stroke protection. Increased edema formation was recently reported in the inflamed skin of *Vasp^−/−^* mice [18] and hypoxia has been shown to foster vascular leakage by downregulation of VASP *in vitro* [28]. Therefore, we injected mice the vascular tracer Evan's Blue to investigate whether VASP is also involved in stroke-induced BBB damage and edema formation. Evan's Blue staining of the brain parenchyma was absent in healthy *Vasp^−/−^* and *Vasp* ^+/+^ mice as well as sham-operated controls of either genotype suggesting that VASP is of minor importance for the regulation of vascular permeability under basal conditions (not shown). 24h after tMCAO BBB leakage, i.e. Evan's Blue extravasation was more pronounced in *Vasp^−/−^* mice compared with littermate controls (39.1 mm^3^±17.2 mm^3^ versus 19.5 mm^3^±7.9 mm^3^, respectively; p<0.05) (Figure 2C). Accordingly, *Vasp^−/−^* mice developed significantly more brain edema (0.8 mm^3^±0.4 mm^3^ versus 1.7 mm^3^±0.5 mm^3^, respectively; p<0.0001).

## Discussion

We here demonstrate that VASP is crucial for maintaining vascular integrity in the ischemic brain. *Vasp* deficiency resulted in enhanced BBB disruption, edema formation and neuronal damage after experimental stroke in mice.

There is accumulating evidence that VASP is critically involved in stabilizing endothelial barriers. Several studies could demonstrate that VASP co-localizes with cell-cell contacts (e.g. the tight junction marker zonula occludens protein-1) and focal adhesion sites (e.g. VE-cadherin) in endothelial cell cultures [15], [18], [19]. Linkage of intercellular contacts and focal adhesions to the intracellular actin cytoskeleton is important for sufficient sealing [29]. VASP has been shown to regulate actin organization at cadherin-adhesive contacts [30] and stabilize endothelial barrier function by promoting actin polymerization and relaxation of the actin cytoskeleton [18], [19]. Consequently, transendothelial permeability was significantly increased in endothelial cells from *Vasp^−/−^* mice [15], [18] and Ena/VASP triple null mice [20].

Most of the data underscoring the significance of VASP for preserving vascular integrity have so far been derived from *in vitro* studies which in addition were mainly conducted in microvascular endothelial cell lines from myocardium, lung or skin under physiological conditions [15], [16], [19], [28]. We here confirm and further extend these findings by demonstrating that VASP also prevents BBB damage and edema formation in the brain after tMCAO in mice, a well established *in vivo* model of ischemic stroke. Hypoxemia is a potent trigger of vascular leakage [31]–[33] and downregulation of VASP by hypoxia-inducible factor (HIF) possibly participates in this process at least in cell culture systems [28]. Because HIF is also strongly induced in the murine brain after tMCAO [34]–[36], HIF-dependent degradation of VASP could also be functionally relevant for edema formation in ischemic stroke *in vivo*. Interestingly, inhibition of HIF-1 using small interfering RNA reduced Evan's Blue extravasation and brain ischemia-reperfusion injury in rats [36]. Two recent *in vitro* studies investigated a possible implication of VASP for hypoxia-induced BBB disruption. Davis and co-workers found that hypoxia-induced VEGF expression increased BBB permeability and correlated with VASP phosphorylation, which was in part mediated through the VEGF receptor 2 [37]. In the second study, the subcellular distribution of VASP in immortalized brain endothelial cells was significantly altered under hypoxic conditions [38].

Inflammation is another well-established trigger of increased vascular permeability [12]. In a model of LPS-induced acute lung injury, *Vasp^−/−^* mice showed increased pulmonary damage, neutrophil infiltration and vascular leakage compared with wild-type animals [39]. Benz and co-workers [18] recently reported that *Vasp^−/−^* mice develop more skin edema upon subcutaneous injections of the proinflammatory peptide hormone bradykinin, the end product of the kallikrein/kinin-system. In line with these findings, we [40], [41] and others [42] could show that endogenous bradykinin also fosters edema formation in ischemic stroke and traumatic brain injury. However, the exact molecular interplay between the kallikrein/kinin-system and VASP in the context of hypoxia, inflammation and vascular leakage needs to be further established.

Taken together, our study indentifies VASP as critical regulator of BBB maintenance and fluid hemostasis during cerebral ischemia. Interference with VASP or VASP-dependent signalling pathways could become a promising strategy to treat excessive brain edema in stroke and possibly other neurological diseases afflicted with severe BBB disruption.

## Materials and Methods

### Animals

A total of 89 mice were used in this study. Animal experiments were approved by legal state authorities (Bezirksregierung of Unterfranken, approval number 54-2531.01-25/06) and conducted according to recent recommendations for research in basic stroke studies including blinded evaluation of the results, randomization of animals, predefinition of exclusion criteria, and power calculations (see below) [43]. The generation and extensive characterization of *Vasp^−/−^*mice is described elsewhere [44]. 6–8 week old male and female *Vasp^−/−^*mice were used and wild-type (WT) littermates (*Vasp^+/+^)* matched for age and sex served as controls.

### Stroke model

The transient middle cerebral artery occlusion (tMCAO) model was applied to induce focal cerebral ischemia as described elsewhere [45], [46]. Briefly, mice were anesthetized with 2% isoflurane in a 70% N_2_O/30% O_2_ mixture. A servo-controlled heating blanket was used to maintain core body temperature close to 37°C throughout surgery. Following a midline neck incision a standardized silicon rubber-coated 6.0 nylon monofilament (60-1720RE; Doccol, Redlands, CA, USA) was inserted into the right common carotid artery and advanced via the internal carotid artery to occlude the origin of the MCA. After 60 min mice were re-anesthetized and the occluding filament was removed to allow reperfusion. Operation time per animal did not exceed 15 min and operators (PK, CK and MA) were blinded for the respective genotypes throughout the study.

The exclusion criteria were as follows:

- Death within 24h after tMCAO
- Subarachnoid hemorrhage (SAH; as macroscopically assessed during brain sampling)
- Bederson score (see below)  = 0 (24 h after tMCAO)

3 out of 43 WT mice (6.9%) and 4 out of 46 *Vasp^−/−^* mice (8.7%) met at least one of the exclusion criteria (2 deaths and 1 non-fatal SAH in the WT group and 3 deaths and 1 non-fatal SAH in the *Vasp^−/−^* group, respectively). The excluded animals were used only for mortality analysis (Figure 2B). 82 out of 89 mice (92.1%) were included for final analysis.

### Anatomical assessment of the cerebral vasculature

For assessment of the cerebral vasculature *Vasp*-deficient mice and controls (n = 5/group) were deeply anesthetized with CO_2_ and transcardially perfused with 4% paraformaldehyde (PFA), followed by 3 ml black ink diluted in 4% PFA (1∶5 v/v). Brains were carefully removed, fixed in 4% PFA overnight at 4°C and the Circle of Willis and major arteries were examined under a microscope. To further quantitatively examine the vascular structures, we graded the development of the posterior communicating arteries (PComAs), which can affect brain sensitivity to ischemia [23], according to the following score: 0, absent; 1, capillary anastomosis; 2, small truncal vessel; 3, patent.

### Regional cerebral blood flow measurement

Laser-Doppler flowmetry (Moor Instruments, U.K.) was used to monitor regional cerebral blood flow (rCBF) in *Vasp^−/−^* mice and WT controls before surgery (baseline), immediately after MCA occlusion, and 10 minutes after removal of the occluding monofilament (reperfusion) (n = 5/group) [47]. For this procedure a small incision was made in the skin overlying the temporal muscle, and a 0.7 mm flexible laser-Doppler probe (model P10) was positioned perpendicular on the superior portion of the temporal bone (6 mm lateral and 2 mm posterior from bregma). This position corresponds to the core of the ischemic territory.

### Invasive hemodynamics

For invasive hemodynamics *Vasp^−/−^* mice and controls (n = 4/group) were anesthetized with 2.5% isoflurane and catheterized via the right carotid artery with a high-fidelity 1.4 F Millar microtip catheter as described [40]. Hemodynamic data (blood pressure and heart rate) were digitized via a MacLab system (AD Instruments, Castle Hill) connected to an Apple G4 PowerPC computer and analyzed.

### Assessment of functional outcome

After recovery from anesthesia and again after 24h, neurological function was assessed by two investigators unaware of the genotype according to the Bederson score (n = 15 in the wild-type and n = 17 in the *Vasp^−/−^*group, respectively) [48] with: 0, no deficit; 1, forelimb flexion; 2, as for 1, plus decreased resistance to lateral push; 3, unidirectional circling; 4, longitudinal spinning; 5, no movement. In addition, the mortality rate was monitored until 24h after tMCAO.

### Determination of infarct size and edema volumes

Animals were sacrificed 24h after tMCAO. Brains were quickly removed and cut in three 2-mm thick coronal sections using a mouse brain slice matrix (Harvard Apparatus, Holliston, MA, USA). The slices were stained for 20 min at 37°C with 2% 2,3,5-triphenyltetrazolium chloride (TTC; Sigma-Aldrich, Taufkirchen, Germany) in PBS to visualize the infarctions [46], [49].

Direct, i.e. without correction for brain edema, and indirect, i.e. corrected for brain edema, infarct volumes (n = 15 in the wild-type and n = 17 in the *Vasp^−/−^*group, respectively) were calculated by volumetry (ImageJ software, National Institutes of Health, USA) according to the following equations:



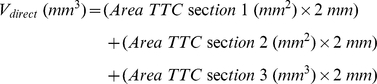





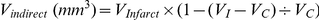



where the term 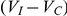
represents the volume difference between the ischemic hemisphere and the control hemisphere and 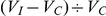 expresses this difference as a percentage of the control hemisphere.

Brain edema volumes (n = 15 in the wild-type and n = 17 in the *Vasp^−/−^*group, respectively) were then calculated by subtracting indirect from direct infarct volumes.

### Determination of blood-brain-barrier leakage

To determine the permeability of the cerebral vasculature 2% Evan's Blue tracer (Sigma Aldrich, Germany) diluted in 0.9% NaCl was i. v. injected 2h after the induction auf tMCAO [40]. After 24h mice (n = 5/group) were transcardially perfused with 4% PFA and brains were quickly removed and cut in 2-mm thick coronal sections using a mouse brain slice matrix (Harvard Apparatus, Holliston, MA, USA). Volumetric measurements (ImageJ software, National Institutes of Health, USA) of the brain parenchyma stained by Evan's Blue were performed to estimate the extent of BBB damage. Sham-operated mice in which the occluding filament was not inserted into the MCA (n = 3/group) and healthy animals (n = 3/group) served as controls.

### Statistics

Results are expressed as mean ± standard deviation (SD). For statistical analysis, PrismGraph 4.0 software package (La Jolla, CA, USA) was used. Data were tested for Gaussian distribution with the D̀Agostino and Pearson omnibus normality test and then analyzed by the unpaired, two-tailed Student's t-test, except for the Bederson score which was analyzed by the non-parametric Mann Whitney test. Mortality rates (Figure 2A) were compared by the Fisher̀s exact contingency test. For the comparison of regional cerebral blood flow (Figure 1B), Bonferroni-corrected 2-way ANOVA was applied. P-values <0.05 were considered to be statistically significant.

For power and type-II (beta) error calculations on infarct volumes GraphPad Stat Mate 2.0 software package was used (GraphPad Software, Inc, La Jolla, CA, USA): On day 1 after 60 min tMCAO the mean infarct volume was 39.3±10.7 mm^3^ in *Vasp^+/+^* mice and 56.1±17.3 mm^3^ in *Vasp^−/−^* mice (Figure 2A). We assumed that a reduction or increase in infarct size of ≥35% (Delta  = 13.8 mm^3^) would be of biological relevance [50], [51]. The significance level (alpha) was chosen as 0.05 (two-tailed). The group size was n = 15 for WT mice and n = 17 for *Vasp^−/−^* mice. Given those premises the power to detect a difference of 13.8 mm^3^ between the mean infarct volumes reached 75% in our study (type-II [beta] error of 25%, respectively) which is a robust result compared to many other experimental stroke studies [43], [52].
